# Mutations in tubulin genes are frequent causes of various foetal malformations of cortical development including microlissencephaly

**DOI:** 10.1186/2051-5960-2-69

**Published:** 2014-07-25

**Authors:** Catherine Fallet-Bianco, Annie Laquerrière, Karine Poirier, Ferechte Razavi, Fabien Guimiot, Patricia Dias, Laurence Loeuillet, Karine Lascelles, Cherif Beldjord, Nathalie Carion, Aurélie Toussaint, Nicole Revencu, Marie-Claude Addor, Benoit Lhermitte, Marie Gonzales, Jelena Martinovich, Bettina Bessieres, Maryse Marcy-Bonnière, Frédérique Jossic, Pascale Marcorelles, Philippe Loget, Jamel Chelly, Nadia Bahi-Buisson

**Affiliations:** Université de Montréal –CHU Sainte Justine, Montréal, QC Canada; Pathology Laboratory, Rouen University Hospital, Rouen, France; NeoVasc Region-Inserm Team ERI28, Laboratory of Microvascular Endothelium and Neonate Brain Lesions, Institute of Research for Innovation in Biomedicine, University of Rouen, Rouen, France; Institut Cochin, Université Paris-Descartes, CNRS (UMR 8104), Paris, France; Inserm, U1016 Paris, France; Département de Génétique, AP-HP, hôpital Necker-Enfants Malades, Paris, France; Institut Imagine, Université Paris Descartes - Sorbonne Paris Cités, Paris, France; Institut Imagine-INSERM UMR-1163, Embryology and genetics of congenital malformations, Paris, France; Pathology Laboratory, Hopital Robert Debré APHP, Paris, France; Clinical Genetic Department, Hospital de Santa Maria, Lisboa, Portugal; Pathology Laboratory, Hopital Poissy St Germain en Laye, Paris, France; Children’s Neurosciences Department, Evelina London Children’s Hospital London, London, UK; Service de Biologie Moléculaire et Genetique, Pavillon Cassini AP-HP, Hôpital Cochin, Paris, France; Centre de Génétique Humaine UCL, Cliniques universitaire Saint Luc, Bruxelles, Belgique; Génétique Médicale-Maternité, CH-1011 CHUV Lausanne Suisse, Lausanne, Suisse; Service d’anatomopathologie, Hôpitaux Universitaires de Strasbourg, Nouvel Hôpital Civil, Strasbourg Cedex, France; Service de Génétique et d’Embryologie Médicales, Université Pierre et Marie Curie, Paris 6, UK; Unit of Fetal Pathology, AP-HP, Hospital Antoine Béclère, Paris, France; Services d’Anatomopathologie et de Génétique, CHU de Nantes, France; Service d’Anatomie Pathologique, CHU, Hôpital Morvan, Brest, France; Service d’anatomie et cytologie pathologiques Hôpital Pontchaillou – Université de Rennes 1 – CHU, Rennes, France

**Keywords:** Microlissencephaly, Lissencephaly, Polymicrogyria, Microcephaly, Tubulin genes

## Abstract

Complex cortical malformations associated with mutations in tubulin genes are commonly referred to as “Tubulinopathies”. To further characterize the mutation frequency and phenotypes associated with tubulin mutations, we studied a cohort of 60 foetal cases. Twenty-six tubulin mutations were identified, of which *TUBA1A* mutations were the most prevalent (19 cases), followed by *TUBB2B* (6 cases) and *TUBB3* (one case). Three subtypes clearly emerged. The most frequent (n = 13) was microlissencephaly with corpus callosum agenesis, severely hypoplastic brainstem and cerebellum. The cortical plate was either absent (6/13), with a 2–3 layered pattern (5/13) or less frequently thickened (2/13), often associated with neuroglial overmigration (4/13). All cases had voluminous germinal zones and ganglionic eminences. The second subtype was lissencephaly (n = 7), either classical (4/7) or associated with cerebellar hypoplasia (3/7) with corpus callosum agenesis (6/7). All foetuses with lissencephaly and cerebellar hypoplasia carried distinct *TUBA1A* mutations, while those with classical lissencephaly harbored recurrent mutations in *TUBA1A* (3 cases) or *TUBB2B* (1 case). The third group was polymicrogyria-like cortical dysplasia (n = 6), consisting of asymmetric multifocal or generalized polymicrogyria with inconstant corpus callosum agenesis (4/6) and hypoplastic brainstem and cerebellum (3/6). Polymicrogyria was either unlayered or 4-layered with neuronal heterotopias (5/6) and occasional focal neuroglial overmigration (2/6). Three had *TUBA1A* mutations and 3 *TUBB2B* mutations. Foetal *TUBA1A* tubulinopathies most often consist in microlissencephaly or classical lissencephaly with corpus callosum agenesis, but polymicrogyria may also occur. Conversely, *TUBB2B* mutations are responsible for either polymicrogyria (4/6) or microlissencephaly (2/6).

**Electronic supplementary material:**

The online version of this article (doi:10.1186/2051-5960-2-69) contains supplementary material, which is available to authorized users.

## Introduction

Disorders of cerebral cortical development are generally classified according to the developmental stage the lesions are observed. Recently, an updated classification that takes into account genetic abnormalities as well as disrupted process and stage of brain development has been proposed. This classification system divides brain malformations into disorders of neuronal and glial proliferation, neuronal migration, and post-migrational development [1]. In living patients, malformations of the cerebral cortex, such as microcephaly, classic lissencephaly or polymicrogyria, represent a major cause of neurodevelopmental disability and epilepsy. In recent years, significant progress has been made into the understanding of underlying genetic bases of these conditions ((see for review [1]). However, in severe cases, death occurs in infancy or early childhood, and in affected foetuses accurate phenotypic descriptions and molecular diagnosis have only rarely been established. In the last 10 years, major progress has been made in the recognition of such malformations, especially through the use of foetal magnetic resonance imaging (MRI) [2, 3]. Ventriculomegaly, prominent subarachnoid spaces, corpus callosum agenesis, and a small head circumference may indicate abnormal brain development and should lead to referral of patients to foetal neurology centers. Additional ultrasound findings suggestive of cortical malformations include either the presence of antenatally appearing abnormal, overdeveloped sulci and gyri, delayed appearance of landmark sulcation or abnormally thin cortex. Foetal brain imaging and particularly MRI is a very useful tool for detecting, and confirming abnormal cortical development. MRI and ultrasound investigations have led to major advances in the classification of these disorders [4–8]. However, these advances need to be completed by studies focused on malformation-associated tissue architecture, and more importantly, definition of genetic causes and specific developmental pathways involved in the constitution of the lesions.

Recent genetic evidence has pointed out to the critical effects of tubulins in regulating neuronal migration. Currently, mutations in tubulin genes encoding different α- and β-tubulin isotypes (*TUBA1A*, *TUBA8* and *TUBB2B, TUBB3, TUBB5*) and very recently γ-tubulin (*TUBG1*) have been involved in a large spectrum of developmental brain disorders affecting living patients, who present with variable degrees of intellectual deficiency, motor delay, seizures and microcephaly. MRI demonstrates a wide spectrum of brain dysgenesis ranging from simplified gyral pattern to agyria, resembling the classical lissencephaly but in combination with specific features which are not observed in *ARX, LIS1, DCX, or RELN* mutations [9–31].

Only few foetal case reports are presently available in the literature, all but one having been reported by our group. Of these, six carried *TUBA1A* mutations, one *TUBB2B* mutation, and one *TUBB3* mutation [12, 23, 25, 32, 33]. Although foetal cases collectively represent the most severe end of the tubulinopathy spectrum, they exhibit various cortical abnormalities, ranging from multifocal polymicrogyria to microlissencephaly with almost absent cortical plate. Histological features undoubtedly provide insights into the pathophysiology underlying these complex cortical developmental malformations. The recognition of the genetic origin of these brain malformations is essential for an appropriate genetic counseling in these families, particularly for subsequent pregnancies. Therefore, by studying a large cohort of 26 foetuses with mutations in tubulin genes, the aim of the present study is to describe the detailed neuropathology and the specific features allowing for the diagnosis of foetal brain tubulinopathies. Our data and conclusions demonstrate that tubulinopathies are frequently implicated in foetal complex malformations of the cortical development, particularly regarding microlissencephalies. In this cohort, *TUBA1A* mutations represent the major cause of microlissencephaly, although *TUBB2B* and *TUBB3* mutations may also be found. The additional major finding is that *TUBA1A* mutations are responsible for a wide spectrum of foetal brain malformations ranging from the most severe; microlissensencephaly to classical lissencephaly, and in some cases to polymicrogyria. By contrast, *TUBB2B* mutations mostly account for generalized or multifocal polymicrogyria.

## Materials and methods

### Patient selection

As part of our ongoing genetic and molecular investigations of patients and families with cortical malformations, DNA samples of 60 foetuses were referred to our laboratory for molecular screening after termination of the pregnancy. Of the 60 patients, 7 were previously reported by our group and re-evaluated for the purpose of this review, 5 with *TUBA1A* mutations, one with *TUBB2B* mutations, one with *TUBB3* mutations [17, 23, 25, 31–33].

In all cases, brain anomalies had been detected on routine ultrasound examination during the second trimester of pregnancy, subsequently confirmed by MRI. These anomalies included ventricular enlargement, either isolated or associated with agenesis of corpus callosum and/or cerebellar anomalies. The cerebellum was described as cystic or with a Dandy–Walker malformation-like appearance. Pregnancies were terminated with the informed consent of the parents and in accordance with the French law. Data regarding family history and foetal/antenatal clinical ultrasound (US) examinations were obtained in all cases.

### Molecular analyses

For genetic and molecular investigations, informed consent was obtained from both parents in all cases. Molecular screening was performed on genomic DNA extracted from frozen foetal tissue, according to standardized protocols. Mutation analysis of the coding regions of the 6 tubulin genes *TUBA1A*, *TUBA8*, *TUBB2B*, *TUBB3*, *TUBB5* and *TUBG1* was carried out on all patients, as previously described [15, 17, 20, 23, 25, 28]. For all patients found to be mutated in tubulin genes, parental DNA was analyzed by direct sequencing to assess the *de novo* occurrence of the mutations.

### Autopsy procedures

All cases but 3 underwent a complete autopsy performed by foetopathologists according to standardized protocols, including X-rays, photographs, and macroscopic and histological examination of all viscera. Foetal biometric data were assessed according to the morphometric criteria of Guihard-Costa et al. [34]. Twenty out of 26 foetuses were personally re-evaluated by C.F.B. Three were initially examined by one of the expert neuropathologists (F.R or A.L) and therefore not re-examined. Concerning the three remaining patients, parents had refused post-mortem examination, so that these foetuses were diagnosed and evaluated on the basis of foetal MRI performed at 30 weeks of gestation (WG) only.

### Neuropathological examination

Brains were fixed in a 10% buffered formalin-zinc solution for 3 to 6 weeks. Brain growth was evaluated according to the biometric criteria of Guihard-Costa and Larroche [35]. The hemispheres, brainstem and cerebellum were cut in a coronal plane, except for some cases in which a medial sagittal plane was carried out in order to examine in more detail vermian abnormalities. At least 4 to 5 serial sections involving one or both hemispheres, 3 to 5 serial axial sections of brainstem and cerebellum or one sagittal section of the brainstem and vermis were embedded in paraffin, cut at either 7 μm (brainstem and cerebellum) or 8 to 10 μm (hemispheres) and stained with hemalun-phloxin, cresyl violet or cresyl violet-luxol fast blue (Klüver-Barrera staining).

The diagnosis of cortical dysgenesis was made on routine histology, and included 3 patterns of lesions: microlissencephaly in 28 cases, lissencephaly in 14 and either typical or atypical polymicrogyria in 18 cases.

## Results

### Mutations

**Figure 1:**
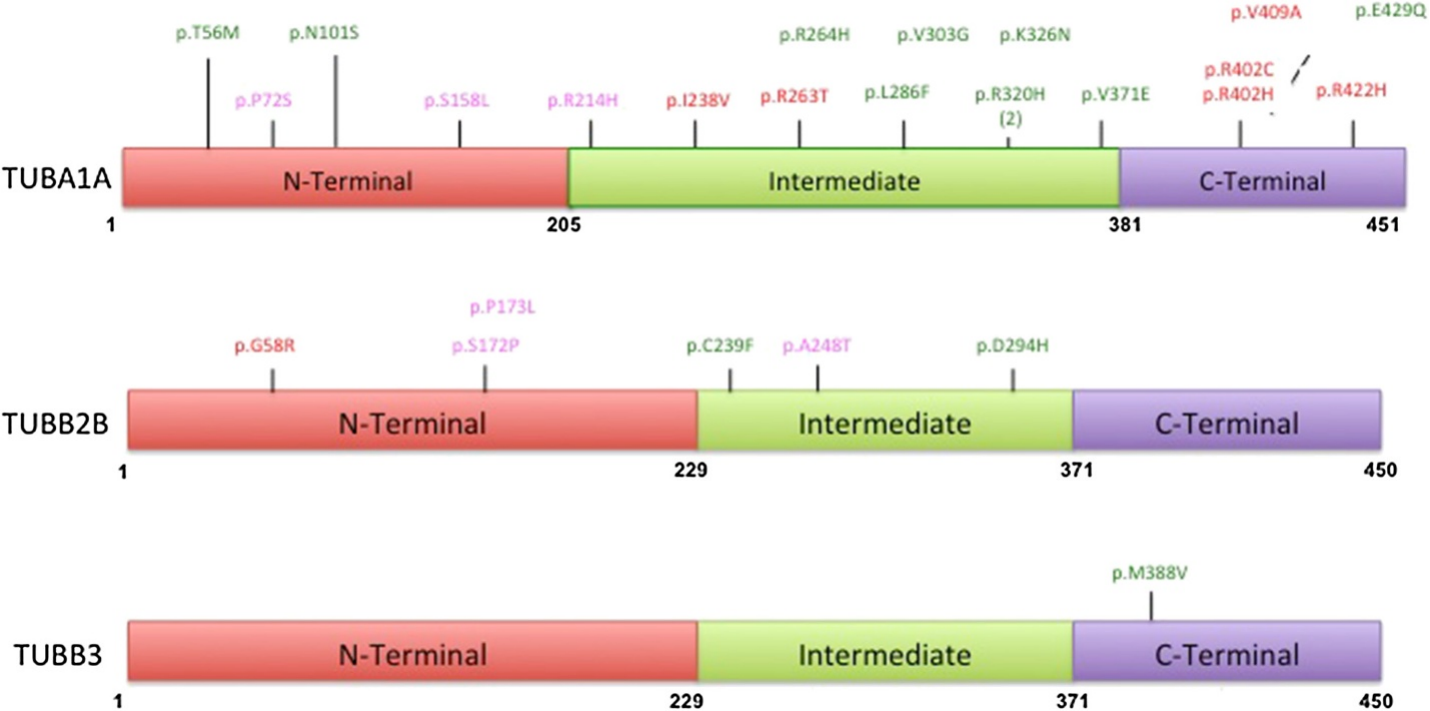
**Schematic representation of the functional domains of TUBA1A, TUBB2B and TUBB3 tubulin subunits and distribution of mutations associated with foetal cases with malformations of cortical development.** Illustrated domains are the N-terminal that contains the guanine nucleotide-binding region, intermediate domain, and C-terminal domains that constitutes the binding surface for MAPs and molecular motors such as kinesins and dyneins. In β-tubulin, they correspond to residues 1–229, 230–371, and 372–450, [36] and in α-tubulin, to residues 1–205, 206–381, 382–451 [37], respectively. Mutations associated with a lissencephaly (classical and with cerebellar hypoplasia) phenotype are indicated in red, with microlissencephaly in green, with polymicrogyria-like cortical dysplasia in pink. For recurrent variations the number of occurrences is indicated in brackets.

Genetic and molecular investigations of foetal cases with complex malformations of cortical development allowed us to identify *TUBA1A, TUBB2B* and *TUBB3* mutations in 26 out of the 60 cases (43.3%) referred to our laboratories (Cochin Hospital and Cochin Institute Laboratories). Of these, we found 19 *TUBA1A*, 6 *TUBB2B* and 1 *TUBB3* mutations. All mutations were different missense mutations, and were shown to occur *de novo*. Of these 26, 19 are newly reported. The 7 foetuses that had been previously reported were reanalysed for the purpose of the study (see for detailed results Figure 1 and Tables 1, 2 and 3).

**Table 1:** **Neuropathology overview of foetuses interrupted for tubulin related microlissencephaly**

Case number	***Gene***	Gender	Nucleotidic change	Proteic change	TOP	Cortical lamination	Neuronoglial overmigration	Heterotopia	Olfactory bulbs agenesis	Hippocampus	Enlarged GZ	Basal Ganglia	Corpus callosum	Cerebellum	Pons –Braintem	Ophthalmological signs	Head circumference	Additional morphological signs	Reference
															Nuclei and corticospinal tract				
LIS_TUB_008_ fœtus13	*TUBB2B*	M	c.716G > T	p.C239F	*16*	Absent CP (2 layers including molecular layer)	Focal	-	N/A	N/A	+	N/A	c.ACC	Severe Hypoplasia and Dysplasia	Severe pons hypoplasia	N/A	<3rd p	N/A	This series
LIS_TUB_004_ fœtus08	*TUBA1A*	M	c.978A > C	p.K326N	*23*	Thick 2-layered cortex	-	+	+	N	+	Hypoplasic	c.ACC	Severe Hypoplasia and Dysplasia	Severe pons hypoplasia	-	<3rd p	Microretrognatism	This series
															Absent CST				
LIS_TUB_006_ fœtus04	*TUBA1A*	M	c.856C > T	p.L286F	*23*	Poorly differentiated CP (2–3 layers poorly individualized)	-	-	+	N	+	Hypoplasic	c.ACC	Moderate Hypoplasia	Severe pons hypoplasia	-	<3rd p	Hypoplastic external genital organs	[32]
															Severe hypoplasia of the CST				
LIS_TUB_080_ fœtus24	*TUBA1A*	F	c.1112 T > A	p.V371E	23,3	Poorly differentiated CP (2–3 layers poorly individualized)	-	-	N/A	Non individualized	+	-	c.ACC	Severe Hypoplasia	Severe pons hypoplasia with hypoplasic olivary nuclei	-	<3rd p	Microretrognatism	This series
															Severe hypoplasia and Disorganization of the CST				
LIS_TUB_002_ fœtus20	*TUBA1A*	F	c.790C > T	p.R264H	24	Absent CP (2 layers including molecular layer)	-	Radial columnar heterotopic neurons	+	N	+	+	c.ACC	Moderate Hypoplasia	Severe pons hypoplasia with fragmented olivary nuclei	-	<3rd p	Microretrognatism	
															Severe hypoplasia and Disorganization of the CST				
LIS_TUB_003_ fœtus18	*TUBA1A*	M	c.167 C > T	p. T56M	*24,3*	Absent CP (2 layers including molecular layer)	-	+	-	Non individualized	+	Hypoplasic	c.ACC	Severe Hypoplasia	Severe pons hypoplasia with hypoplasic olivary nuclei	Optic Nerve Hypoplasia	<3rd p	Microretrognatism	This series
															Severe hypoplasia and Disorganization of the CST				
LIS_TUB_004_ fœtus09	*TUBA1A*	F	c.1285G > C	p.E429Q	*25*	4 layered cortex	-	Radial columnar heterotopic neurons	-	Non individualized	+	Hypoplasic	c.ACC	Severe Hypoplasia	Severe pons hypoplasia with hypoplasic olivary nuclei	-	<3rd p	Microretrognatism/dysmorphy/Exophthalmia/Hypertelorism	This series
															Severe hypoplasia and Disorganization of the CST				
LIS_TUB_005_foetus01	*TUBA1A*	M	c.959G > A	p.R320H	*25*	Absent CP	Focal	Radial columnar heterotopic neurons	-	Non individualized	+	-	p.ACC	Severe Hypoplasia and Dysplasia	Severe hypoplasia (neuronal overmigration)-spinal cord anterior horn hypoplasia	N/A	<3rd p	Microretrognatism/dysmorphy/Exophthalmia/Hypertelorism	This series
															Absent CST				
LIS_TUB_079_ fœtus25	*TUBA1A*	M	c.302A > G	p.N101S	25	Poorly differentiated CP (2–3 layers poorly individualized)	-	Dispersed heterotopic neurons	+	Non individualized	N/A	-	c.ACC	Severe Hypoplasia and Dysplasia	Severe pons hypoplasia with hypoplasic olivary nuclei	-	<3rd p	Microretrognatism/dysmorphy/Exophthalmia/Hypertelorism	This series
															Severe hypoplasia and Disorganization of the CST				
LIS_TUB_009_ fœtus19	*TUBB2B*	M	c.745G > C	p.D294H	*27*	Absent CP (2 layers including molecular layer)	Massive -	-	-	Non individualized	+	-	c.ACC	Severe Hypoplasia	Severe hypoplasia with focal overmigration	N/A	<3rd p	Absent	This series
															Absent CST				
LIS_TUB_010_ fœtus17	*TUBB3*	M	c.1162A > G	p.M388V	*27*	Thin CP with 2 layers	-	Dispersed heterotopic neurons	+	N	+	Hypoplasic	c.ACC	Severe Hypoplasia and Dysplasia	Severe pons hypoplasia	Optic Nerve Hypoplasia	<3rd p	Absent	[33]
															Severe hypoplasia of the CST				
LIS_TUB_006_ fœtus03	*TUBA1A*	F	c.908 T > G	p.V303G	*36*	Thick severe 2 layered	-	-	-	N	+	Hypoplasic	p.ACC	Severe Hypoplasia and Dysplasia	Severe pons hypoplasia	-	<3rd p	Absent	[33]
															Severe hypoplasia of the CST				
LIS_TUB_081_ fœtus26	*TUBA1A*	M	c.959G > A	p.R320H	*26*	Absent CP (2 layers including molecular layer)	-	Dispersed and nodular heterotopic neurons	N/A	N/A	-		c.ACC	Severe Hypoplasia and Dysplasia	Severe pons hypoplasia	-	<3rd p	Facial dysmorphism	
															Severe hypoplasia of the CST				

*Abbreviations*: *TOP* termination of the pregnancy, *MicroLis* microlissencephaly, *N/A* not available, *ACC* corpus callosum agenesis, *GZ* germinal zones, *p.* percentile; Foetal MRI based analysis of the phenotype; unilat: unilateral; +: Present; −: Absent; N: Normal; CP: cortical plate; CST: Corticospinal tract.

**Table 2:** **Neuropathological overview of foetuses interrupted for tubulin related lissencephalies**

Case number	***Gene***	Gender	Nucleotidic change	Proteic change	TOP	Cortical lamination	Neuronoglial overmigration	Heterotopia	Olfactory bulbs agenesis	Hippocampus	Enlarged GZ	Basal Ganglia	Corpus callosum	Cerebellum	Pons –Braintem	Ophthalmological signs	Head circumference	Additional morphological signs	Reference
															Nuclei and corticospinal tract				
LIS_TUB_025 fœtus06	*TUBA1A*	M	c.787C > A	p.P263T	*26*	Poorly differentiated CP (2–3 layers poorly individualized)	-	Dispersed heterotopic neurons	+	N	+	Dysmorphic	c.ACC	Severe Hypoplasia	Severe pons hypoplasia	-	5th p	Absent	[32]
															Severe hypoplasia and Disorganization of the CST				
LIS_TUB_011_ fœtus23^1^	*TUBA1A*	M	c.1226 T > C	p.V409A	32	N/A	N/A	N/A	N/A	N/A	N/A	N/A	c.ACC	Severe hypoplasia	Severe pons hypoplasia	N/A	5th p	Absent	This series
LIS_TUB_022_ fœtus05	*TUBA1A*	M	c.712A > G	p.I238V	*25*	Poorly differentiated CP (2–3 layers poorly individualized)	-	Nodular heterotopia	-	N	+	Dysmorphic	c.ACC	Moderate Vermian Hypoplasia	Severe pons hypoplasia	N/A	5th p	Absent	[32]
															Unilateral hypoplasia of the CST				
LIS_TUB_018_ fœtus10	*TUBA1A*	F	c.1265G > A	p.R422H	*28*	N/A	N/A	N/A	N/A	N/A	N/A	N/A	c.ACC	Mild Vermian Hypoplasia	Mild pons hypoplasia	N/A	5th p	Absent	This series
LIS_TUB_017 fœtus02^1^	*TUBA1A*	M	c.1205G > A	p.R402H	*29*	N/A	N/A	N/A	N/A	N/A	N/A	N/A	c.ACC	Mild Vermian Hypoplasia	Mild pons hypoplasia	N/A	5th p	Absent	This series
LIS_TUB_013_ fœtus14	*TUBB2B*	F	c.302G > A	p.G98R	*32,8*	Thick 4-layered cortex	-	Heterotopia	N/A	N	N/A	-	c.ACC	Mild Vermian Hypoplasia	Mild pons hypoplasia	-	5th p	Absent	This series
LIS_TUB_021_ fœtus07	*TUBA1A*	M	c.1204C > T	p.R402C	*35*	Thick 4-layered cortex	-	Radial columnar heterotopic neurons	-	N	-	-	Thick CC	Mild Vermian Hypoplasia	Moderate pons hypoplasia	N/A	5th p	Absent	[32]
															Mild hypoplasia of the CST				

*Abbreviations*: *TOP* termination of the pregnancy, *LIS* lissencephaly, *N/A* not available, *ACC* corpus callosum agenesis, *GZ* germinal zones, *p.* percentile; ^1^no foetopathological data available; Foetal MRI based analysis of the phenotype; unilat: unilateral; +: Present; −: Absent; N: Normal; CP cortical plate; CST: corticospinal tract.

**Table 3:** **Neuropathology overview of foetuses interrupted for polymicrogyria like cortical dysplasia**

Case number	***Gene***	Gender	Nucleotidic change	Proteic change	TOP	Cortical lamination	Neuronoglial overmigration	Heterotopia	Olfactory bulbs agenesis	Hippocampus	Enlarged GZ	Basal Ganglia	Corpus callosum	Cerebellum	Pons –Braintem Nuclei and Corticospinal tract	Ophthalmological signs	Head circumference	Additional morphological signs	Reference
LIS_TUB_012_ fœtus22	*TUBA1A*	F	c.214 C > T	p.P72S	37,8	Unlayered Generalized and Asym PMG (fronto-central predominant)	-	Nodular Heterotopia	N/A	N	N/A	N/A	HypoCC	Severe	Severe pons hypoplasia	N/A	5th p	Absent	This series
														Vermian Hypoplasia					
															Mild hypoplasia of the CST				
LIS_TUB_043_foetus11	*TUBA1A*	M	c.641G > A	p.R214H	*23*	Unlayered Central and Asym PMG	Focal	+	unilat	N/A	-	N	c.ACC	Mild Vermian Hypoplasia	Mild dysplasic olivary nuclei	N/A	10th p	Absent	This series
															Unilateral hypoplasia of the CST				
LIS_TUB_048_foetus16	*TUBB2B*	M	c.742G > A	p.A248T	*28,5*	Unlayered Central and Asym-multifocal PMG	-	Dispersed heterotopic neurons	+	N/A	N/A	N	N	Mild Vermian Hypoplasia	Normal		5th p	Absent	This series
LIS_TUB_053_foetus21	*TUBA1A*	F	c.473C > T	p.S158L	24,5	Unlayered Generalized and AsymPMG	-	Radial columnar heterotopic neurons	+	Dysmorphic	+	Hypoplasic	c.ACC	Severe Hypoplasia and Dysplasia	Hypoplasia Olivar heterotopia	-	5th p	Absent	This series
															Disorganized CST				
LIS_TUB_054_foetus15	*TUBB2B*	M	c.518C > T	p.P173L	*25*	Unlayered Generalized and AsymPMG (central regions)	-	-	N/A	N/A	N/A	Dysmorphic	c.ACC	Moderate hypoplasia	Severe pons hypoplasia	N/A	<3rd p	Absent	This series
															Disorganized CST				
LIS_TUB_056_foetus12	*TUBB2B*	M	c.514 T > C	p.S172P	*27*	Unlayered Generalized and Asym PMG (fronto-central predominant)	Focal	Radial columnar heterotopic neurons	-	N	-	N	c.ACC	Mild Vermian Dysplasia	Normal	N/A	<3rd p	Absent	[23]
															Disorganized CST				

*Abbreviations*: *TOP* termination of the pregnancy, *PMG* polymicrogyria, *N/A* not available, *ACC* corpus callosum agenesis, *GZ* germinal zones, *p* percentile; foetal MRI based analysis of the phenotype; unilat: unilateral; +: Present; −: Absent; N: Normal; Asym: asymmetrical; CST: Corticospinal tract.

### Neuropathological data

Detailed brain examination allowed us to identify a wide spectrum of cortical malformations that can be divided into three groups despite some overlaps: microlissencephaly, lissencephaly and micropolygyria-like cortical dysplasia.

#### Microlissencephaly

Twelve foetuses (8 males and 4 females, from 16 to 36 WG) displayed a combination of extreme microcephaly, corpus callosum agenesis and lissencephaly. For the remaining 13^th^ case, termination of pregnancy was achieved at 16 WG for absent foetal movements, arthrogryposis and microcephaly. Of the 12 cases, 8 presented with non-specific dysmorphic features including retrognathia and hypertelorism, as well as adducted thumbs, extremely long fingers, and rocker bottom feet, due to poor foetal mobility.

**Figure 2:**
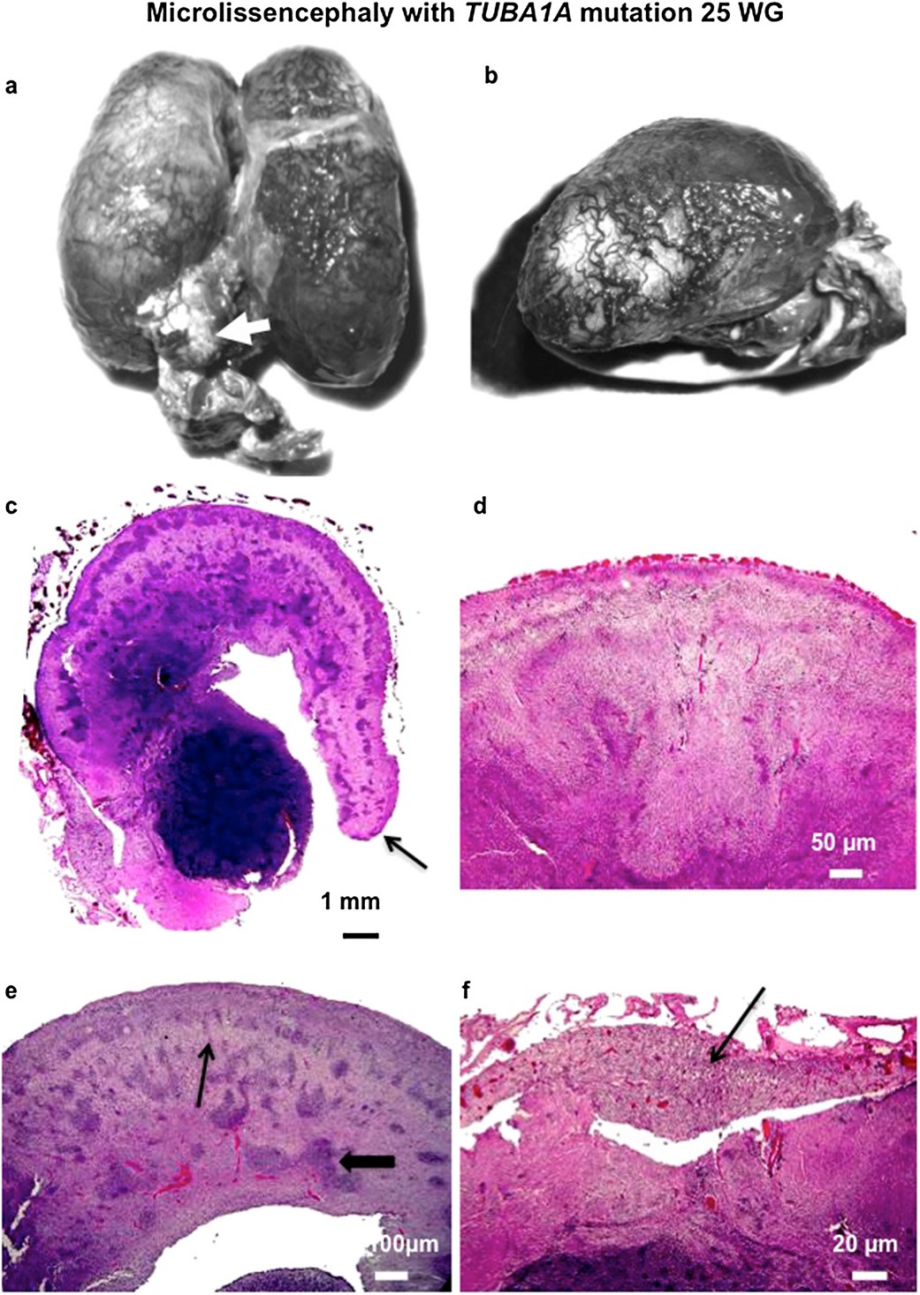
**Hallmarks of microlissencephaly in a 25 WG foetus (LIS_TUB_005_foetus01) with** ***TUBA1A*** **mutation Macroscopical data with abnormally short agyric hemispheres, severe hypoplastic brain stem and cerebellum (white arrow) (a), Smooth brain surface with no sylvian fissure (b), Smooth brain surface with agenesis of the corpus callosum without Probst bundles (arrow), and voluminous germinal zones (c), No individualized cortical plate but a thin layer made up of immature cells is present at the surface of the hemispheres (d), numerous heterotopias either radial (thin arrow) or columnar (thick arrow) in the white matter (e), with focal neuroglial cell overmigration within the meningeal spaces (f), (Scale bars: a, b: 1 mm, c: 50** **μm, d, f: 20** **μm, e: 100** **μm).**

**Figure 3:**
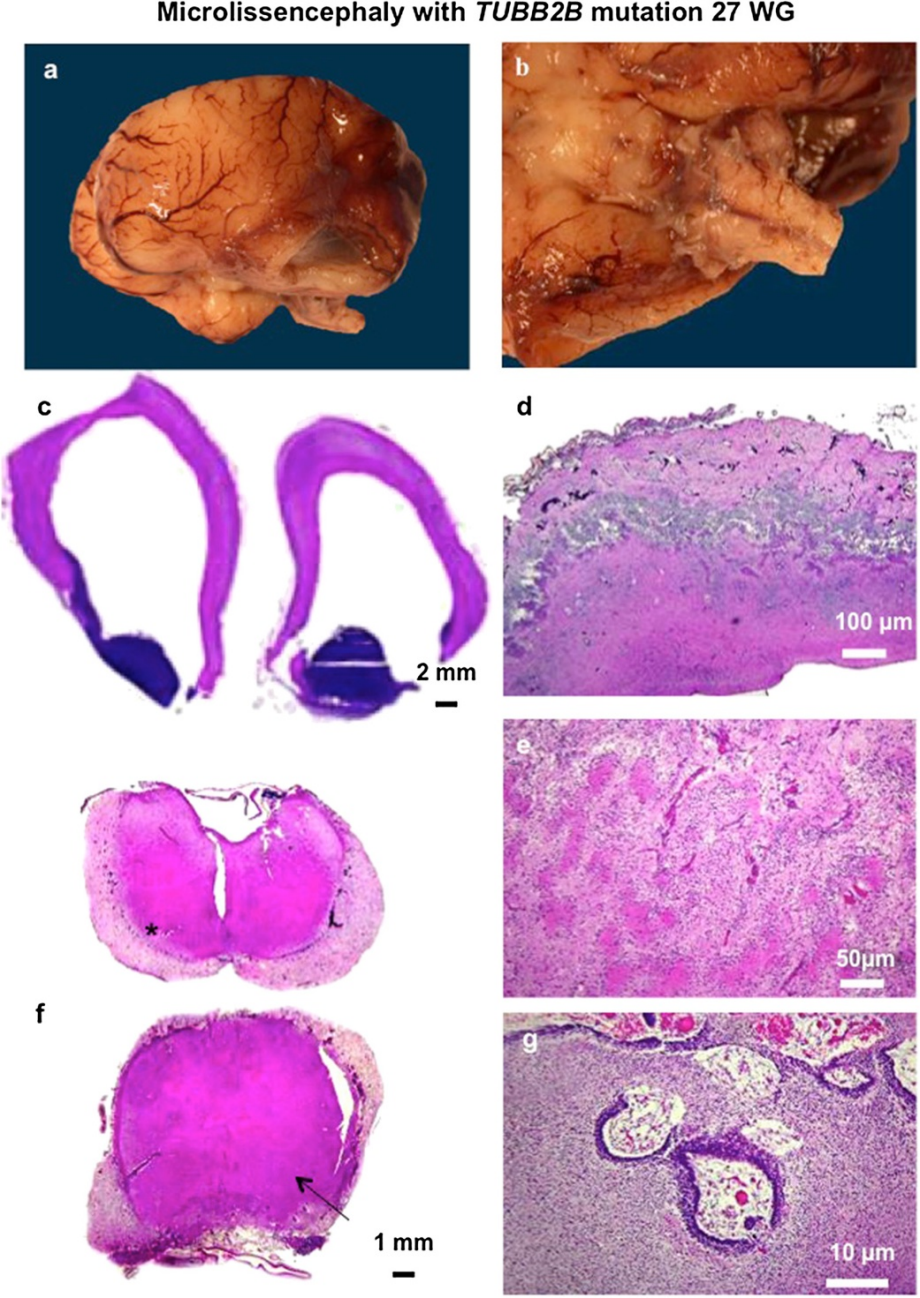
**Macroscopical and histological features of microlissencephaly in a 27 WG foetus (LIS_TUB_009_fœtus19) with** ***TUBB2B*** **mutation.** Macroscopical view of the left hemisphere displaying agyria with absent sylvian fissure absent olfactory bulbs **(a)**; absent olfactory bulbs severely hypoplastic brainstem and cerebellum **(b)**. Coronal section passing through the hemispheres displaying a thin mantle with absent corpus callosum, internal capsule and basal ganglia, along with enlarged ventricles and voluminous germinal zones **(c)**, diffuse disorganization of the cortical plate with massive overmigration of cells within the meningeal spaces **(d)**, with at higher magnification a cobblestone pattern with numerous tortuous vessels **(e)**, thickened meninges containing diffuse overmigration foci at all levels of the brainstem, which is unusually flat, with absent pontine nuclei (arrow) and olivary nuclei (asterisk) **(f)**, disorganized cerebellar cortex cytoarchitecture due to abnormal migration of granule cells in the meninges leading to a fusion of folia identical to what observed in “cobblestone” dysplasia **(g)** (Scale bars: **a**: 2 mm, **b**: 100 μm, **c**, **d**: 50 μm, **e**: 1 mm, **f**: 10 μm).

In all cases, macroscopical examination confirmed consistent features of extreme microcephaly (<3^rd^ percentile) but with no intrauterine growth retardation. The brain surface was completely smooth, lacking primary fissures, olfactory sulci and bulbs (Figures 2a and 3a) and optic nerves in 2 cases. The brainstem and cerebellum were severely hypoplastic, with a widely opened fourth ventricle (Figures 2b and 3b). On sagittal sections, the corpus callosum was absent.

Histological examination revealed severe disturbances of the cortical cytoarchitecture. The cortical plate was either thin (5/13) or with a two-three layered organization made up of a molecular layer, a relatively thin wavy layer with a higher cellular density and a 3^rd^ less cellular layer (Figure 2c). In the most severe cases (6/13), the cortical plate was severely disorganized with a festooned-like pattern and with neither lamination nor clear demarcation between white and grey matter (Figure 3c). These abnormalities radically differ from the 4-layered thick cortex observed in classical lissencephaly. Only two cases demonstrated a thick cortical plate, with 4 layers in one foetus and two layers in the other one.

Moreover, multiple glomerular structures made up of a fibrillar core, surrounded by non-differentiated cells were observed in the deep cortical layers and the underlying subplate, along with whirling heterotopic fascicles within the superficial cortical layer and in the periventricular areas (Additional file 1: Figure S1). Nodular or radial heterotopias were often found within the white matter (8/13) (Figure 2e) and to coexist with neuroglial overmigration foci into the arachnoid space (Figure 3d) (LIS_TUB_005_foetus 1; LIS_TUB_008_foetus 13). An extracortical heterotopic layer responsible for a 'cobblestone-like' cortical dysplasia was observed in a single case (LIS_TUB_009_foetus 19; (Figure 3e,f). Hippocampi were either absent or demonstrated a severely disturbed cytoarchitecture. In all cases but two, histological examination confirmed callosal agenesis with no Probst bundles.

Microlissencephaly was also characterized by its unusual association with voluminous germinal zones and ganglionic eminences exclusively observed in cases of termination of pregnancy during the 2^nd^ trimester (Figures 2c and 3c) (Additional file 1: Figure S1 and Additional file 2: Figure S2). The striatum and pallidum were severely hypoplastic in the majority of the cases or were absent. The internal capsule was also missing, so that the caudate nucleus and putamen appeared to be fused. The thalami were reduced in size and crudely shaped in all cases.

At the infratentorial level, the corticospinal tracts were either absent in most cases, and disorganized in the remaining cases as were the cranial nerve nuclei (except LIS_TUB_005_foetus01 and 17), contrasting with a normal or mildly hypoplastic tectum (LIS_TUB_002_fœtus20). The pontine nuclei were rudimentary with heterotopic neurons most probably destined for the pontine and olivary nuclei (Figure 3f). Remarkably, the foetus (LIS_TUB_009_fœtus19) (Figure 3f) also demonstrated massive neuroglial heterotopia at the level of the brainstem reminiscent of Walker Warburg syndrome. All cases demonstrated global cerebellar hypoplasia with small nodules of heterotopic Purkinje cells in the cerebellar white matter.. In the most severe cases (LIS_TUB_009_fœtus19) (Figure 3 g), the cerebellum was exceedingly hypoplastic with vermian agenesis (Additional file 1: Figure S1 and Additional file 2: Figure S2) Detailed foetopathological features are provided in Table 1.

Overall, the main features of tubulin related microlissencephaly associate severe microcephaly lacking primary fissures, complete corpus callosum agenesis, hypertrophic germinal zones and ganglionic eminences, hypoplasic and disorganized striatum and thalami and severe cerebellar and brainstem hypoplasia.

Most patients with microlissencephaly (10/13) carried mutations in *TUBA1A* gene. Seven are novel, identified in 6 different cases (p.T56M, p.N101S, p.R264H, p.K326N, p.V371E, p.E429Q) except for one mutation (p.R320H) identified in two foetuses, and in two previously reported (p.L286F [32] and p.V303G [33]). Other patients carried respectively distinct mutations in *TUBB2B* (p.C239F [23] and the novel mutation, p.D294H) or *TUBB3B* gene (p.M388V) respectively [25]. Of interest, none were found in other tubulinopathies, either in foetuses or living patients.

#### Lissencephaly

**Figure 4:**
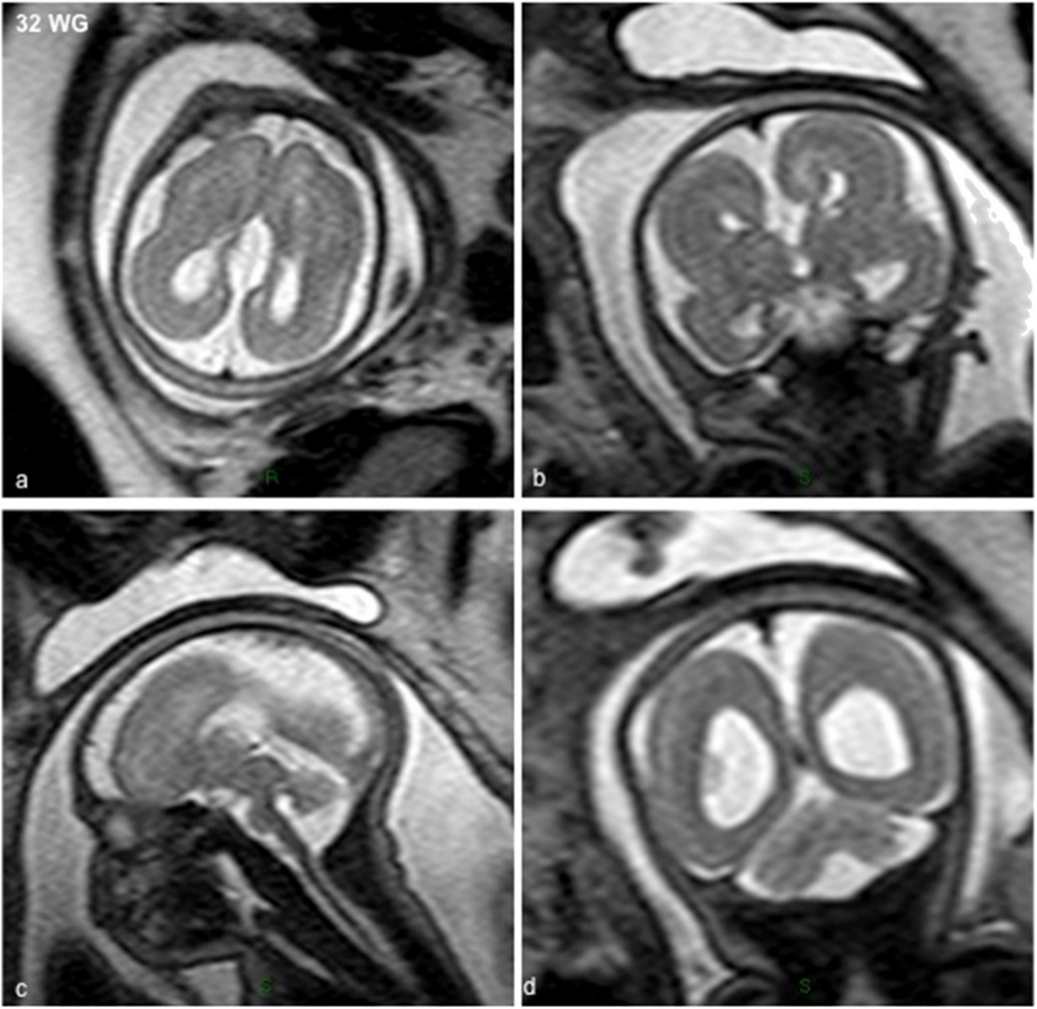
**MRI of LIS_TUB_011 foetus 23 with lissencephaly with cerebellar hypoplasia at 32 WG with** ***TUBA1A*** **mutation (p.V409A) showing complete agyria, virtually no sulci on axial (a) and coronal (b,d) T2-weighted sections, complete corpus callosum agenesis and pontocerebellar hypoplasia on sagittal (c) and coronal (d) sections.**

Seven patients (5 males and 2 females) were referred for a prenatal diagnosis of lissencephaly between 25 to 35 WG. All had reduced biometric brain parameters between 5^th^ and 10^th^ percentile without microcephaly and none were dysmorphic. In all cases, ventriculomegaly detected on ultrasound examination was confirmed on foetal brain MRI performed between 29 and 30 WG (Figure 4) which revealed in addition to ventriculomegaly, lissencephaly associated with corpus callosum agenesis in 6/7 patients and with moderate to severe vermian hypoplasia in 3/7 patients. Based on the MRI features, 3 had a pattern of lissencephaly with cerebellar hypoplasia (LCH), while 4 had features rather compatible with the diagnosis of classical lissencephaly. Four were available for foetopathological examination.

On macroscopic examination, the brain surface was completely smooth with absent Sylvian fissures. Olfactory sulci and bulbs were present in 2/3 foetuses and optic nerves were normal in all cases. Two cases showed severe (LIS_TUB_025 fœtus06) or moderate (LIS_TUB_022_fœtus05) vermian and brainstem hypoplasia, while in the two others, the vermis was mildly hypoplastic [32]. Cortical plate abnormalities were of variable severity, abnormally thick (LIS_TUB_021_fœtus07; LIS_TUB_013_fœtus14) with reduced white matter in 2 cases, and thin in the 2 other cases. On microscopic examination, cortical cytoarchitecture displayed severe lesions. In two cases (LIS_TUB_021_fœtus07 and LIS_TUB_021_ fœtus07), a 4-layered cortex was observed as in LIS1-related lissencephaly, consisting of an upper molecular layer, a second thin cellular layer containing pyramidal neurons usually observed in layer V, a third pale poorly cellular layer and a fourth thick deep layer made up of neurons which had failed to migrate. Heterotopic neurons with a radial columnar organization were found in the deepest part of the cortical mantle. At the infratentorial level, only the vermis was mildly hypoplastic and malrotated, the cerebellar hemispheres contained large Purkinje cell heterotopias. In the medulla, cortico-spinal tracts were flattened, spreading out ventrally and laterally. Olivary nuclei were absent with voluminous olivary heterotopias, as observed in LIS1-related lissencephaly.

The two other cases displayed a 2-layered lissencephaly with a well-identified upper molecular layer and a second thin layer containing mainly immature neurons and only few differentiated pyramidal cells in its deepest part. In one of the 2 cases, the cortical plate consisted of an upper molecular layer and a thicker wavy like cellular layer containing mostly immature neurons [32]. In both cases, heterotopic neurons and glomerular structures were associated with numerous abnormal fascicles either intercrossed or turned back on themselves located in the deep white matter and the periventricular area. At the infratentorial level, (LIS_TUB_022_fœtus05, LIS_TUB_025 fœtus06) the ventral part of the brainstem was more severely affected, with hypoplastic pontine nuclei and corticospinal tracts. In the medulla, the pyramids were absent and olivary nuclei hypoplastic with voluminous olivary heterotopias, reminiscent of LCH.

Neuronoglial overmigration within the leptomenigeal spaces was never found in these cases. In the youngest cases, the germinal zone was voluminous and the basal ganglia, as well as the anterior arm of the internal capsule contained numerous misoriented small fibre bundles. The thalami were also hypoplastic and disorganized. Detailed foetopathological features are provided in Table 2.

The majority of patients with classical lissencephaly (4/7) or with LCH (3/7) also carried mutations in *TUBA1A* gene (6/7). All were different, with one novel (p.V409A) and 5 previously reported (p.R402C [16], p.R402H, p.I238V, p.P263T, and p.R422H [32]). Only one patient with classical lissencephaly (LIS_TUB_013_ fœtus14) carried a *TUBB2B* mutation (p.G98R).

#### Polymicrogyria-like cortical dysplasia

**Figure 5:**
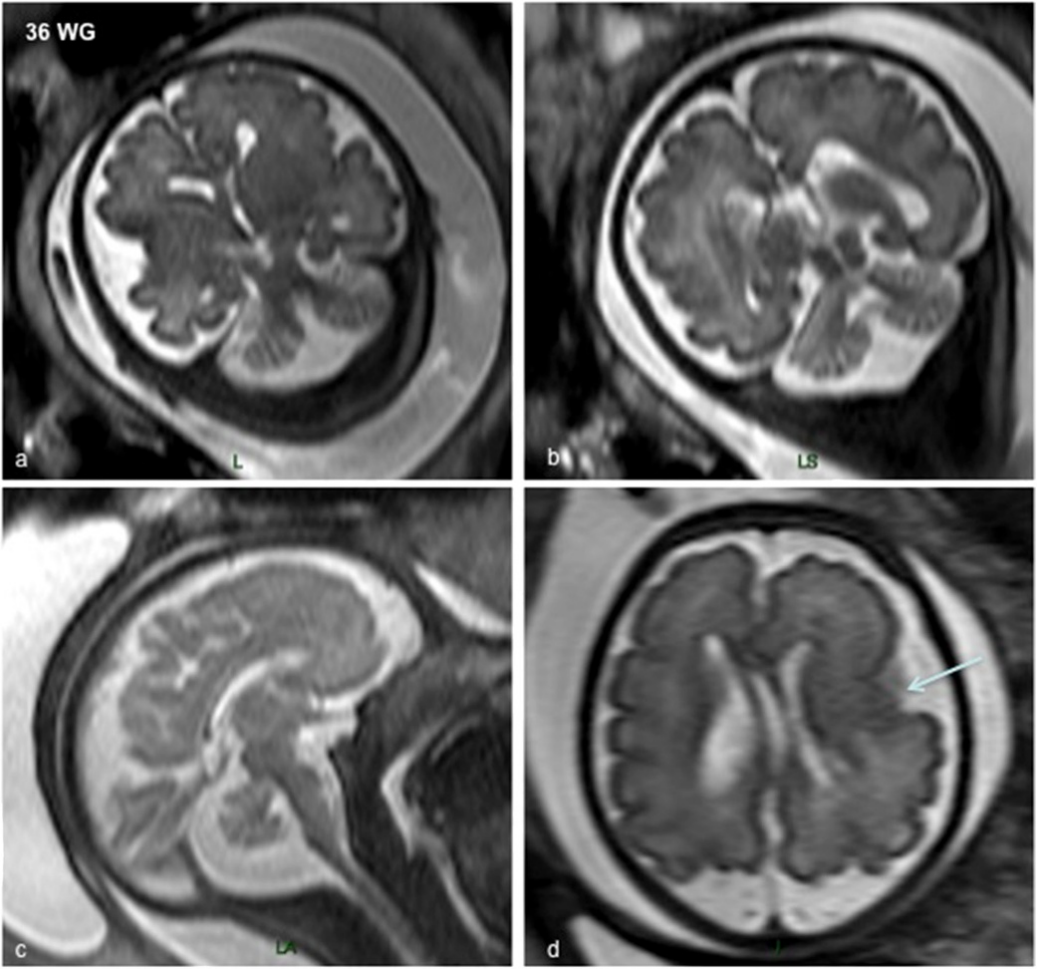
**MRI of LIS_TUB_012 foetus 22 with polymicrogyria-like cortical dysplasia at 36 WG with** ***TUBA1A*** **mutation (p.P72S) showing asymmetrical left predominant perisylvian polymicrogyria on coronal (a,b) and axial (d) T2-weighted sections, the corpus callosum is hypoplastic and thin, and the cerebellum and the brainstem appear to be hypoplastic on sagittal (c) and coronal section (b).**

**Figure 6:**
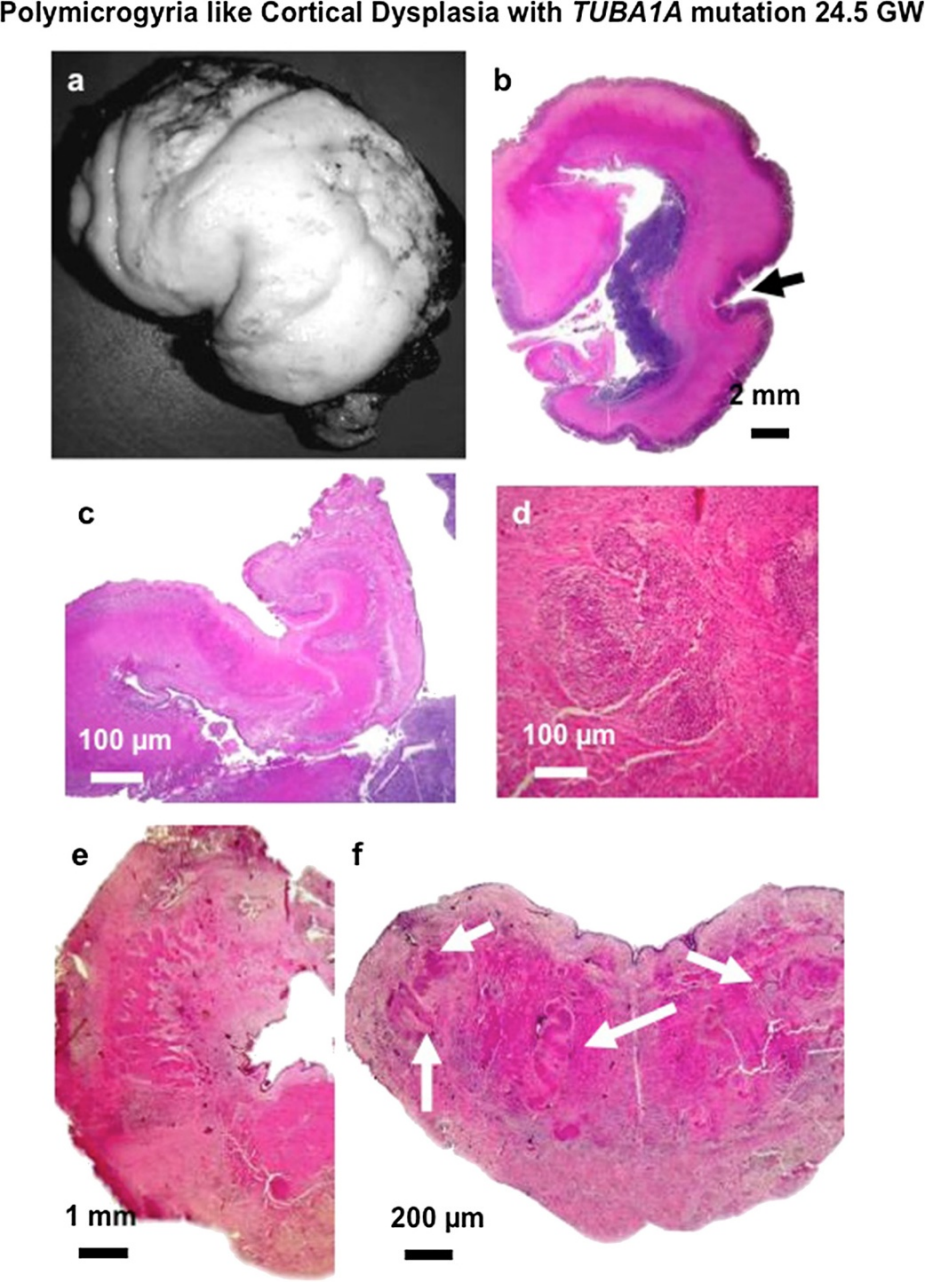
**Macroscopical and histological data in a 24.5 WG foetus (LIS_TUB_053_foetus21) with polymicrogyria-like cortical dysplasia and** ***TUBA1A*** **mutation.** Fronto-parietal polymicrogyria with short and vertically oriented sylvian fissure and cerebellar hypoplasia **(a)**, on coronal sections, enlarged germinal zone with polymicrogyria involving the frontal, perisylvian and temporal areas (arrow) **(b)**, malrotated and hypoplastic hippocampus **(c)**, scattered nodular heterotopias in the deep white matter **(d)**, roughly shaped dentate nuclei **(e)**, absent olivary nuclei with bilateral, large olivary heterotopias found in the dorsal part of the medulla (white arrows) **(f)** (Scale bars: **b**, 2 mm, **c**, **d**: 100 μm, **e**: 1 mm, **f**: 200 μm).

In 6 patients (4 males and 2 females), a polymicrogyric pattern (Figures 5 and 6a,b and Additional file 3: Figure S3) was diagnosed between 23 and 36 WG. In cases diagnosed earlier (respectively 23, 24.5 and 25 WG), imaging features consisted of corpus callosum agenesis and cerebellar and brainstem hypoplasia, while in the others, abnormal gyration was identified on MRI (Figure 5 LIS_TUB012_foetus22). Brain biometric parameters ranged from the 5^th^ and the 10^th^ percentile in 4 cases, and below the 3^rd^ percentile in two cases.

Polymicrogyria represented the less severe end of the spectrum with a multifocal asymmetrical polymicrogyric pattern. On macroscopic examination, the spectrum of gyral abnormalities ranged from asymmetrical abnormal coarse perisylvian gyri with largely opened sylvian fissures to diffuse (4/6) polymicrogyria-like cortical dysplasia (Figure 6a). In all cases, they appeared to predominate in fronto-central regions and never resemble the “morocco leather” pattern classically described in typical polymicrogyria. In addition, foetuses exhibiting generalized polymicrogyria-like cortical dysplasia had more severe cerebellar hemispheric and/or vermian hypoplasia and dysplasia, compared with those with fronto-centrally predominant malformations, which had milder cerebellar hypoplasia. At last, unilateral olfactory bulb agenesis was noted in a single case.

On histological examination, focal cortical anomalies were restricted to the depth of the sylvian fissure in two cases, and were more extensive in the four other cases, involving the frontal, temporal and the parieto-occipital cortices. “Polymicrogyria” was mainly unlayered in all cases, but sometimes intermixed with a 4-layered pattern. In two cases, neuroglial overmigration foci were observed, covering the unlayered polymicrogyria (Additional file 3: Figure S3). By contrast, no overmigration was observed in 4-layered polymicrogyric areas and/or in the normal laminated cortical regions. Hippocampus cytoarchitecture was disorganized in one case (Figure 6c). Of note, the glomerular structures usually observed in the other types of foetal tubulinopathies were identified only in one out of the re-analyzed 4 cases. Abnormal intercrossed fascicles were found in the subthalamic and sublenticular regions in all cases (Figure 6d). Heterotopic neurons, either radial (Figure 6d) or nodular were not seen in combination in the same region. Callosal agenesis was associated with Probst bundles in all cases except for one (LIS_TUB_012_foetus22). Only one case (25 WG) displayed enlarged germinal zones, but these were less voluminous than observed in microlissencephaly and lissencephaly cases, and the caudate nucleus and putamen appeared to be fused, due to absent the internal capsule. In other cases, germinal zones and internal capsule were present, but multiple and short axonal fascicles were observed within the pallidum and under the putamen.

At the infratentorial level, the brainstem was hypoplastic and abnormally flat, with poorly developed pontine nuclei, absent olivary nuclei and large bilateral olivary heterotopias in case of polymicrogyria-like cortical dysplasia (Figure 6f). Corticospinal tracts were aberrantly located. In the cerebellum, the dentate nuclei were abnormally shaped and several large Purkinje cell heterotopias were present in the white matter (Figure 6e). In less severely affected cases, the vermis was moderately hypoplastic without any significant histological changes. In the cerebellar hemispheres, several heterotopias were identified and the dentate nuclei were poorly convoluted and fragmented. Brainstem examination did not reveal any significant lesions. Detailed neuropathological features are provided in Table 3.

Cases with tubulin related polymicrogyria-like cortical dysplasia carried mainly *TUBB2B* mutations (3/6), of which two were novel (p.P173L and p.A248T) and one was the seminal foetal case (p.S172P) [23]. Other three cases carried three novel *TUBA1A* mutations (p.P72S, p.S158L and p.R214H), and very importantly neuroglial overmigration was observed in one of the cases harbouring in *TUBA1A* as well as *TUBB2B* mutations.

## Discussion

We report for the first time a large cohort of foetuses with various malformations of cortical development due to mutations in 3 different tubulin genes (*TUBA1A, TUBB2B* and *TUBB3*), underlining the relatively high frequency of tubulinopathies among the causes of severe complex cortical malformations ranging from polymicrogyria-like cortical dysplasia to microlissencephaly with corpus callosum agenesis and ponto-cerebellar hypoplasia. Although tubulinopathies are now easily suspected after birth by means of neuroimaging studies since their characteristics have been extensively described [9–31], only few foetal cases have been reported so far and significant knowledge of the neuropathology of foetal tubulinopathies is therefore lacking. For these reasons, one of the major strengths of our study was the availability of neuropathological analyses in almost all cases and of the retrospective re-evaluation with the aim of highlighting the key features allowing for the diagnosis. Besides, neuropathological studies remain an invaluable tool for the delineation of novel entities, as well as for their underlying pathophysiological mechanisms.

The different patterns of cerebral involvement and of neuropathological features allowed us to recognize 3 majors groups of cortical dysgenesis [38]: microlissencephaly in which the cortical plate is reduced to a two-layered thin cortex or absent, lissencephaly either classical or variant (thick four-layered cortex with a cell-sparse zone, normal pons and cerebellum, or with either a three or a two-layered cortical plate) and polymicrogyria-like cortical dysplasia.

Until recently, the different possible pathophysiological mechanisms underlying microlissencephaly remained poorly understood. Our study clearly demonstrates that tubulin mutations may represent a significant cause of sporadic microlissencephaly with corpus callosum agenesis. Approximately half of the foetuses with microlissencephaly referred for molecular diagnosis were found to carry mutations mainly in *TUBA1A* gene, and less frequently in *TUBB2B* and *TUBB3* genes. Microlissencephaly is a rare entity characterized by severe congenital microcephaly with absent sulci and gyri leading most of the time to an early fatal outcome during the foetal or the neonatal period. Microlissencephaly was initially considered as belonging to the vast microcephaly spectrum and was thought to result essentially from abnormal neuronal proliferation or survival. Two main microlissencephaly syndromes are recognized: type A, previously called Norman Roberts syndrome [39, 40] and type B also named Barth syndrome. Less than 10 cases with Norman-Roberts syndrome have been described so far. In all cases, microlissencephaly is associated with dysmorphic features consisting of sloping forehead, hypertelorism, broad and prominent nasal ridge and micrognathia [39, 41–43]. In these cases, the neuropathology has been rarely reported, describing either a thin cortical plate with heterotopic neurons [44] or a 4-layered cortex [42]. In both of the reported cases, the corpus callosum was normal and no infratentorial anomalies were observed. In a similar way, few reports of Barth type microlissencephaly are available. This distinct condition is characterized by the association of microlissencephaly with a thin cortical mantle, small thalami, corpus callosum agenesis and of an extreme cerebellar and brainstem hypoplasia [45–48]. Both microlissencephaly type A and B have been observed in consanguineous families suggesting an autosomal recessive inheritance. Conversely, sporadic cases have barely been reported while they represent at least 40% of our foetal cohort, and we have been able to provide evidence here that *TUBA1A* mutations are a major cause of microlissencephaly, accounting for 46.4% of our cases. Moreover, we report for the first time, one *TUBB2B* mutation responsible for microlissencephaly. These results strongly suggest that tubulin mutations should be systematically searched in a context of microlissencephaly with corpus callosum agenesis, particularly when sporadic, starting in order of frequency by *TUBA1A* mutation screening, then *TUBB2* and *TUBB3*. Based on literature review, some previously reported “severe LCH” fall in fact into the microlissencephaly group. For instance, the 5 cases previously reported by Kumar *et al.* (including one foetal case at 21WG) and Cushion *et al*. described the combination of extreme microcephaly, complete corpus callosum agenesis with LCH. Of these, one was due to *TUBB2B* mutation while the remaining 4 were due to *TUBA1A* mutations [12, 29]. This group radically differs from the classical lissencephaly group with cerebellar hypoplasia characterized by diffuse pachygyria but not necessarily by severe microcephaly nor complete corpus callosum agenesis [12]. Because of the potential implications for the genetic screening, our data together with these observations imply that microlissencephaly must be distinguished from LCH, the former being related to either *TUBA1A, TUBB2B* or *TUBB3* mutations, while the latter strongly related to *TUBA1A* mutations.

Our data about foetal tubulinopathies provide new insights into the pathophysiology of lissencephalies and polymicrogyria and strengthen the hypothesis that in the context of tubulin mutations, these two malformations belong to the same spectrum. Recently, polymicrogyria-like cortical dysplasia was proposed to designate the atypical forms of polymicrogyria observed in tubulinopathies, owing to the presence of radial columnar heterotopia and neuronal overmigration through the pia, features that are not typical of most forms of polymicrogyria [29]. Our results reinforce this concept since tubulin-related polymicrogyria display several unusual features. Firstly, the classic macroscopic appearance of “morocco leather” is not present. Secondly, tubulin-related polymicrogyria consists in intermixed unlayered and 4-layered areas, combined with either focal heterotopia or radial columnar heterotopia. Third, focal neuroglial ectopias into the meningeal spaces are often observed, indicating that tubulinopathies result from both abnormal lamination and overmigration through a defective glia limitans. These neuronal overmigrations which represent the hallmark of type II (cobblestone) lissencephaly [1, 49, 50] and were initially described as focal when associated with *TUBB2B* mutations are also a significant feature of tubulin related polymicrogyria and microlissencephaly. In tubulinopathies, neuroglial ectopias may be either focal and mild or massive, with a thick cellular extracortical layer that could be erroneously interpreted as a Walker Warburg syndrome. They are also observed either in a context of microlissencephaly (3 cases) or polymicrogyria (2 cases), and associated with either *TUBA1A* or *TUBB2B* mutations, but are absent in classical lissencephaly. The exact role of tubulins in the establishment of the pia membrane is still poorly understood. It is worth mentioning that these features (the overmigration in the pial membrane observed in fetuses with tubulin mutations) has been previously reported in the cortex and cerebellum of the mouse model of GPR56-related bifronto-parietal polymicrogyria (BFPP) and also in human fetal cases of BFPP associated with mutations in GPR56 [51–53]. Gpr56, an orphan G protein–coupled receptor, localizes to radial glial foot processes directly adjacent to the pial basement membrane and is required to maintain structural integrity of this basement membrane. In vitro investigations have shown that several GPR56 mutations identified in human disrupt the intracellular trafficking of the receptor, which is no longer located in glial-end feet and does not participate in the molecular scaffolding sustaining the basement membrane [54]. The radial glia disruption in TUBA1A and TUBB2B-related malformations of cortical development associated with neuroglial overmigration in meningeal spaces might result from an impaired MT-dependent intracellular trafficking of transmembrane receptors and adhesion molecule normally present in glia-end feet caused by the alteration of microtubule cytoskeleton, a hypothesis that awaits future investigation [55, 56].

In the previously reported tubulin-related lissencephaly cases, the cortical plate was either two-layered with virtually absent normal laminar organization or four-layered, coexisting with a band of ectopic neurons of diverse shape and size organized in columns or clusters, lying in a reduced rim of white matter reminiscent of *LIS1* related lissencephaly. Both patterns strongly differ from neocortical neuronal arrangement observed in the *DCX* related lissencephaly characterized by a “six-layered cortex” with a band of ectopic neurons of varying shape and size organized in columns or clusters, lying in a reduced rim of white matter [57]. In *DCX* mutations, layers I and II of the cortex are well defined, and pyramidal and polymorphic neurons of layers III, IV, V, and VI are found more or less in their appropriate location. This contrasts with the inverted pattern described in *LIS1* and some *TUBA1A* related lissencephalies where large pyramidal neurons of layer V and VI, often inverted, are found in heterotopic positions beneath the superficial molecular layer I [58]. In some cases, histological examination of tubulin-related lissencephaly reveals a quasi absence of lamination, with a two-layered cortex consisting of a layer I with scarce and misplaced Cajal–Retzius cells and a single ill-defined band of neurons extending from the inferior limit of the marginal zone to the periventricular zone [32, 33, 59]. This pattern also significantly differs from foetal *ARX* related lissencephaly, where the cortical plate is mainly three-layered and contains exclusively pyramidal neurons with an absence of interneurons [60]. By contrast, interneurons are found in the cortex in tubulin related lissencephaly, although they may be reduced in number, as in other lissencephalies [32, 61, 62].

It is noteworthy that irrespective of the cortical anomaly, other brain malformations are usually present in tubulin related cortical dysgeneses, consisting of hypoplasic and dysplastic, often fragmented basal ganglia. At the infratentorial level, most tubulin related cortical dysgeneses are associated hypoplastic pons and medulla with indiscernible pontine and brainstem nuclei and absent corticospinal tracts. Olivary nuclei are usually absent with large heterotopia. In the cerebellum, the dentate nuclei are usually fragmented, Purkinje cells are reduced in number in the cerebellar cortex, heterotopically located in the cerebellar white matter and arranged in small clusters or in streaks intermingled with hypoplastic deep cerebellar nuclei. These observations are consistent with the various phenotypes observed in living patients.

Another striking feature observed in foetal tubulinopathies consists in the presence of enlarged ventricular germinal zones and voluminous ganglionic eminences. They are observed not only in all tubulin related microlissencephalies, but also in some lissencephalies and polymicrogyria whatever the mutated gene. These anomalies are observed in foetuses corresponding to pregnancies terminated during the second trimester. Their pathophysiological mechanisms remain still partly unexplained but it is well admitted that about 80%–90% of all cortical neurons originate from the germinal ventricular and subventricular zones and migrate radially to reach their final place in the cortex [63]. From the 5^th^ post-conceptional week, proliferation of neural stem cells in the neuroepithelium thickens the cortical wall of the ventricular zone. Subsequently, with the successive waves of migration toward the cortical surface, the germinal region breaks down during the third trimester of the pregnancy, the ventricular zone and ganglionic eminences disappear progressively and the brain loses its germinal potential [64]. By 25–27 gestation weeks the human ventricular zone has reduced in size to a one-cell-thick ependymal layer [65]. Strikingly, the majority of foetuses with brain tubulinopathies demonstrate the combination of reduced brain parameters and/or microcephaly and hypertrophic germinal zones. These findings may reflect the failure of post-mitotic neuroblasts to initiate their migration toward the cortex leading to a thickening of the ventricular wall and resulting in an accumulation of these cells in the germinal zones and ganglionic eminences with subsequent impaired cortical lamination. The apparent “normalization” of the volume observed in foetal cases interrupted at the end of the gestation might be either due to a delayed migration, to apoptosis of neurons that failed to exit the germinal zones or to both mechanisms. This hypothesis of a transient migration defect and of a delayed migration rather than an arrest of migration is reminiscent of our recent findings regarding the consequences of Tubb3 knockdown on radial migration. Using *in utero*-electroporation experiments, we have shown that Tubb3 knockdown leads to delayed radial migration suggesting that the neuronal arrest is a transient phenomenon, and that neurons that do not express Tubb3 maintain their migratory potential [66]. Finally, this study further confirm the potential implication of tubulins in the regulation of axonal outgrowth, guidance, and differentiation, as reflected by anomalies of the cortico-spinal tracts and corpus callosum, and the presence of small rounded glomerular structures, dystrophic axonal tracts with aberrant directions abnormal whirling heterotopic fascicles often observed in the periventricular white matter [32].

To conclude, the present study demonstrates that tubulinopathies, and more specifically *TUBA1A* mutations, represent one of the major genetic aetiologies of sporadic microlissencephalies. Though informative cellular characterization and phenotyping remain an issue, this study made it possible to describe relevant histopathological findings in details, which in turn provides new insights into the understanding of MRI anomalies observed in patients with tubulin-related malformations of cortical development.

## Electronic supplementary material

Additional file 1: Figure S1: Microscopic findings in 23 WG foetus with microlissencephaly and *TUBA1A* mutation (LIS_TUB_004_ fœtus09). Microlissencephaly associated with abnormally voluminous ganglionic eminences, corpus callosum agenesis and abnormally shaped hippocampi (a), fusion of the putamen and caudate nucleus due to the absence of the anterior limb of internal capsule (b), with Probst bundles (arrow) (c), presence of heterotopic whirling fascicles in the cortical plate (arrow) (d), strongly hypoplastic brainstem and cerebellum with a flattened ventral part of the pons due to hypoplastic pontine nuclei and fragmented dentate nuclei in the cerebellum (e, shown enlarged in g), rudimentary olivary nuclei with almost indiscernible pyramids in the medulla (f). (Scale bars: a: 2 mm, b: 1 mm, c: 200 μm, d: 100 μm, e: 1 mm, f, g: 200 μm). (TIFF 3 MB)

Additional file 2: Figure S2: Histological lesions in 25 WG foetus microlissencephaly and *TUBA1A* mutation (LIS_TUB_003_ fœtus18). Microlissencephaly with a 2-layered cortical plate with reduced white matter restricted to a periventricular rim, and corpus callosum agenesis without Probst bundles (a, boxed area is shown enlarged in b), “wavy” pattern of the superficial layer of the cerebral mantle (c), voluminous ganglionic eminences compared to the overall brain size (d), and neuroglial cell overmigration within the meninges covering the hemispheres (e), severe brainstem and cerebellum hypoplasia, due to absence of corticospinal tracts and pontine nuclei (f). Agenesis of the pyramids and absent olivary with bilateral heterotopias (g). (Scale bars: a: 1 mm, b: 200 μm, c, e: 100 μm, d, f: 1 mm, g: 200 μm). (TIFF 3 MB)

Additional file 3: Figure S3: Histological data in 27 WG foetus with polymicrogyria and *TUBB2B* mutation (LIS_TUB_056_foetus12). On hemispheric coronal sections, histological examination demonstrates a polymicrogyria associated with white matter heterotopias (a, b), radial heterotopias at higher magnification (c), neuroglial cell overmigration associated with polymicrogyria in some limited areas (d) and disorganized cytoarchitecture of right and left hippocampi (e, f). (Scale bars: a, b: 1 mm, e, f, g, h: 100 μm). (TIFF 3 MB)
