# Supplementary figures and images for: Mutations in tubulin genes are frequent causes of various foetal malformations of cortical development including microlissencephaly
Source: Acta Neuropathol Commun. 2014 Jul 25;2:69. doi: 10.1186/2051-5960-2-69 (PMC4222268; doi:10.1186/2051-5960-2-69)

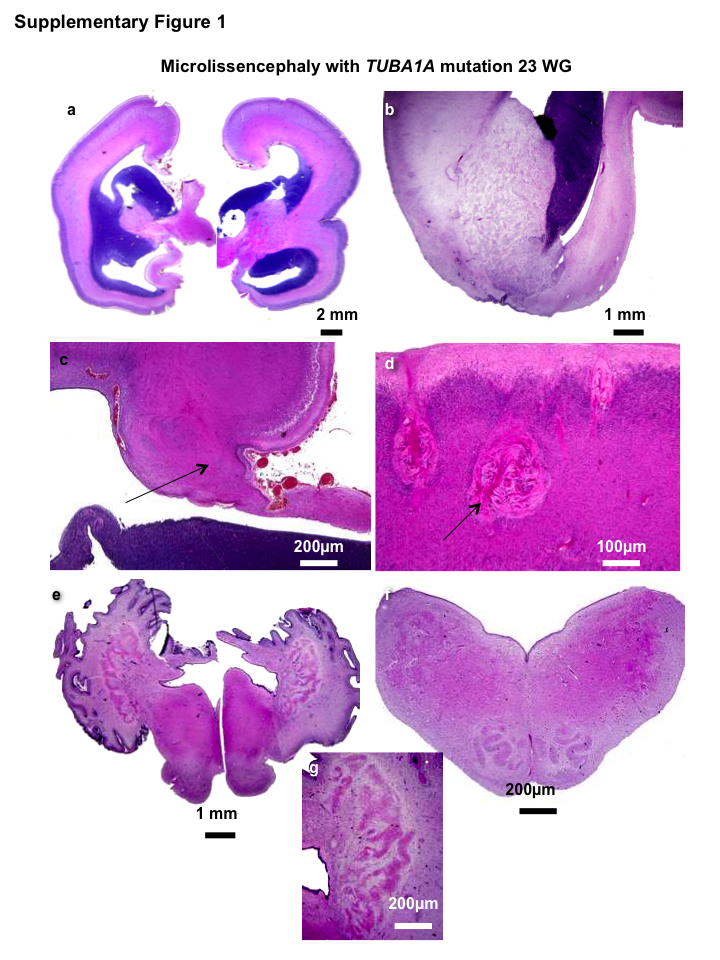

Supplement: Supplementary file 1 — Additional file 1: Figure S1: Microscopic findings in 23 WG foetus with microlissencephaly and TUBA1A mutation (LIS_TUB_004_ fœtus09). Microlissencephaly associated with abnormally voluminous ganglionic eminences, corpus callosum agenesis and abnormally shaped hippocampi (a), fusion of the putamen and caudate nucleus due to the absence of the anterior limb of internal capsule (b), with Probst bundles (arrow) (c), presence of heterotopic whirling fascicles in the cortical plate (arrow) (d), strongly hypoplastic brainstem and cerebellum with a flattened ventral part of the pons due to hypoplastic pontine nuclei and fragmented dentate nuclei in the cerebellum (e, shown enlarged in g), rudimentary olivary nuclei with almost indiscernible pyramids in the medulla (f). (Scale bars: a: 2 mm, b: 1 mm, c: 200 μm, d: 100 μm, e: 1 mm, f, g: 200 μm). (TIFF 3 MB) [file 40478_2014_152_MOESM1_ESM.tiff]

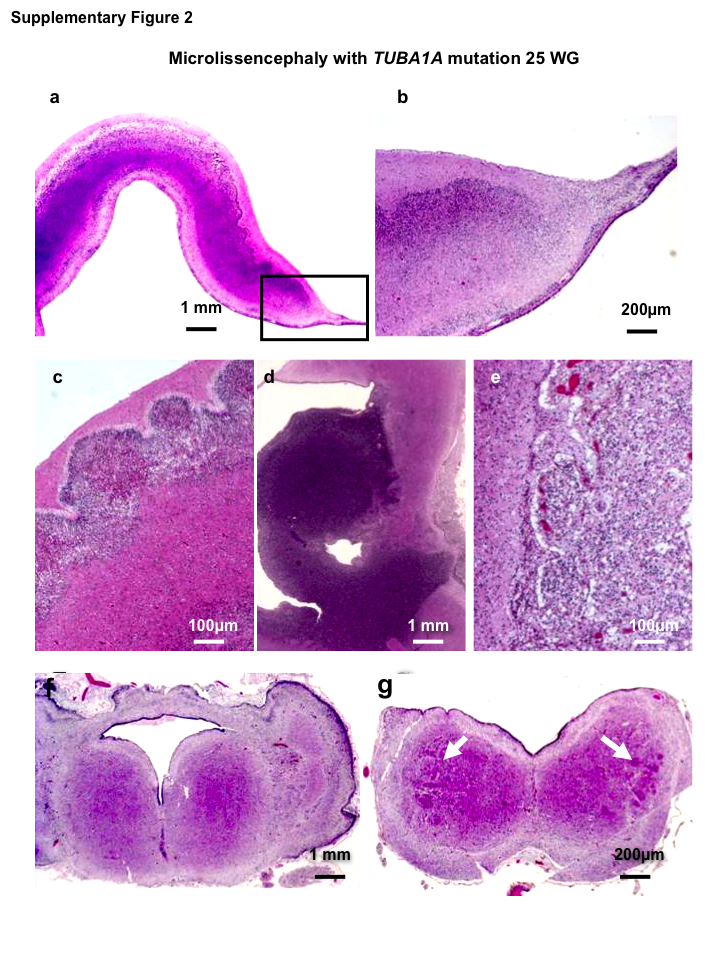

Supplement: Supplementary file 2 — Additional file 2: Figure S2: Histological lesions in 25 WG foetus microlissencephaly and TUBA1A mutation (LIS_TUB_003_ fœtus18). Microlissencephaly with a 2-layered cortical plate with reduced white matter restricted to a periventricular rim, and corpus callosum agenesis without Probst bundles (a, boxed area is shown enlarged in b), “wavy” pattern of the superficial layer of the cerebral mantle (c), voluminous ganglionic eminences compared to the overall brain size (d), and neuroglial cell overmigration within the meninges covering the hemispheres (e), severe brainstem and cerebellum hypoplasia, due to absence of corticospinal tracts and pontine nuclei (f). Agenesis of the pyramids and absent olivary with bilateral heterotopias (g). (Scale bars: a: 1 mm, b: 200 μm, c, e: 100 μm, d, f: 1 mm, g: 200 μm). (TIFF 3 MB) [file 40478_2014_152_MOESM2_ESM.tiff]

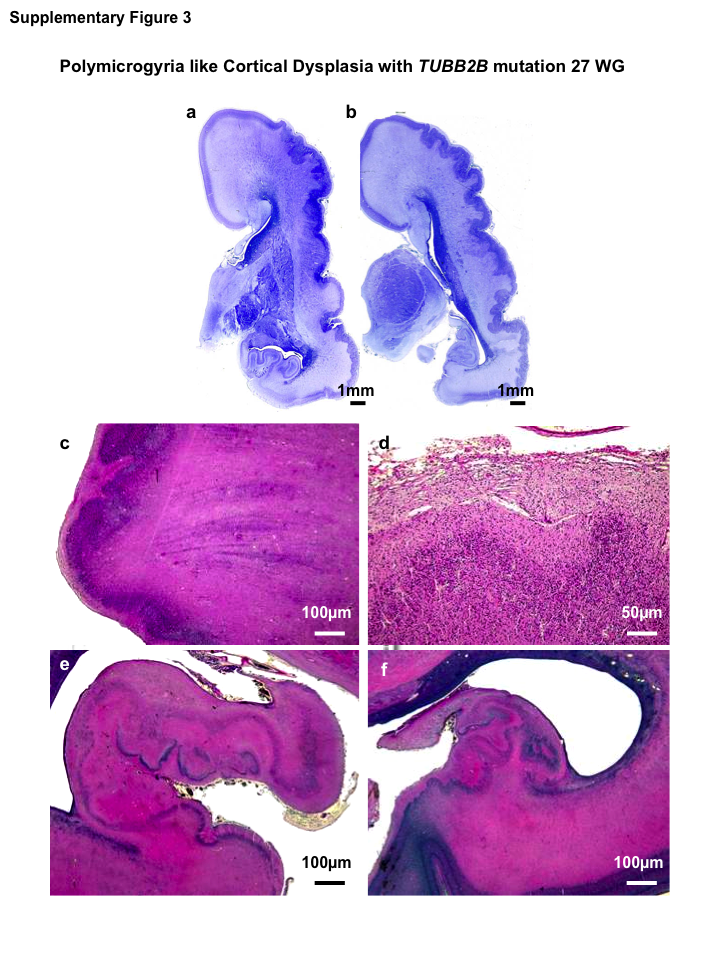

Supplement: Supplementary file 3 — Additional file 3: Figure S3: Histological data in 27 WG foetus with polymicrogyria and TUBB2B mutation (LIS_TUB_056_foetus12). On hemispheric coronal sections, histological examination demonstrates a polymicrogyria associated with white matter heterotopias (a, b), radial heterotopias at higher magnification (c), neuroglial cell overmigration associated with polymicrogyria in some limited areas (d) and disorganized cytoarchitecture of right and left hippocampi (e, f). (Scale bars: a, b: 1 mm, e, f, g, h: 100 μm). (TIFF 3 MB) [file 40478_2014_152_MOESM3_ESM.tiff]
